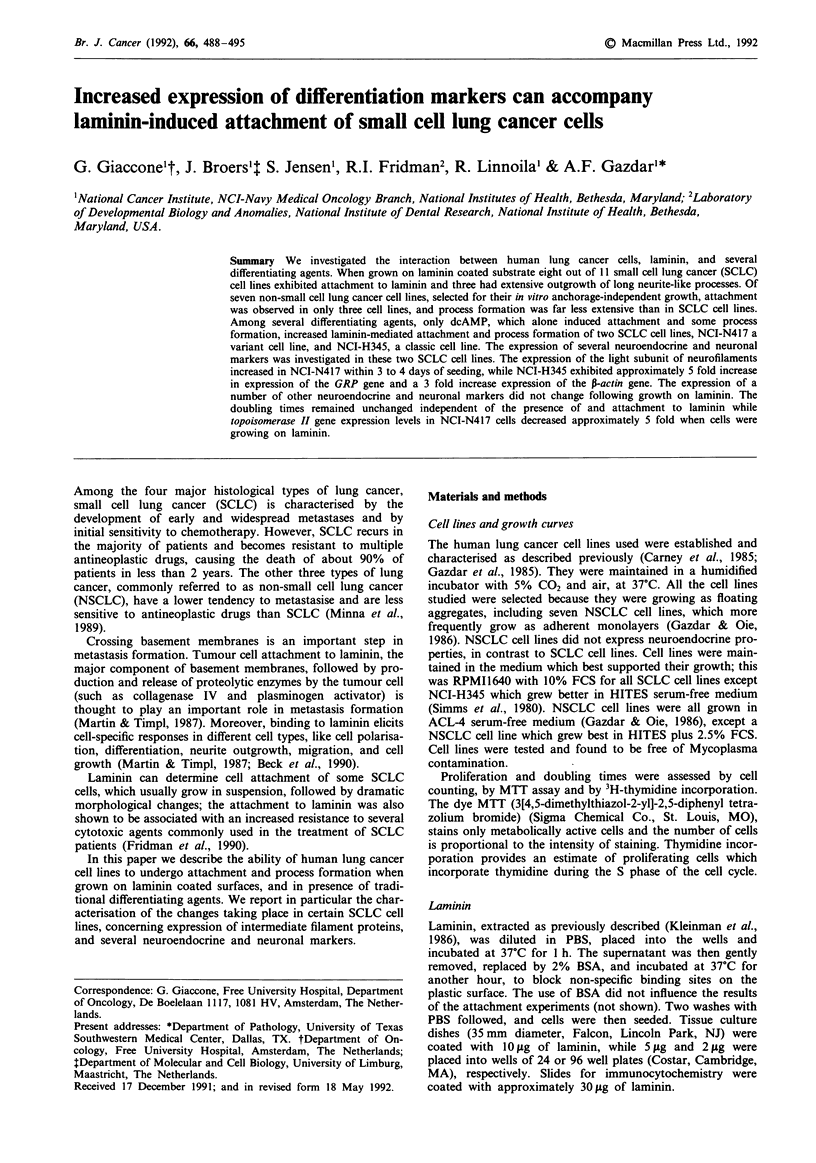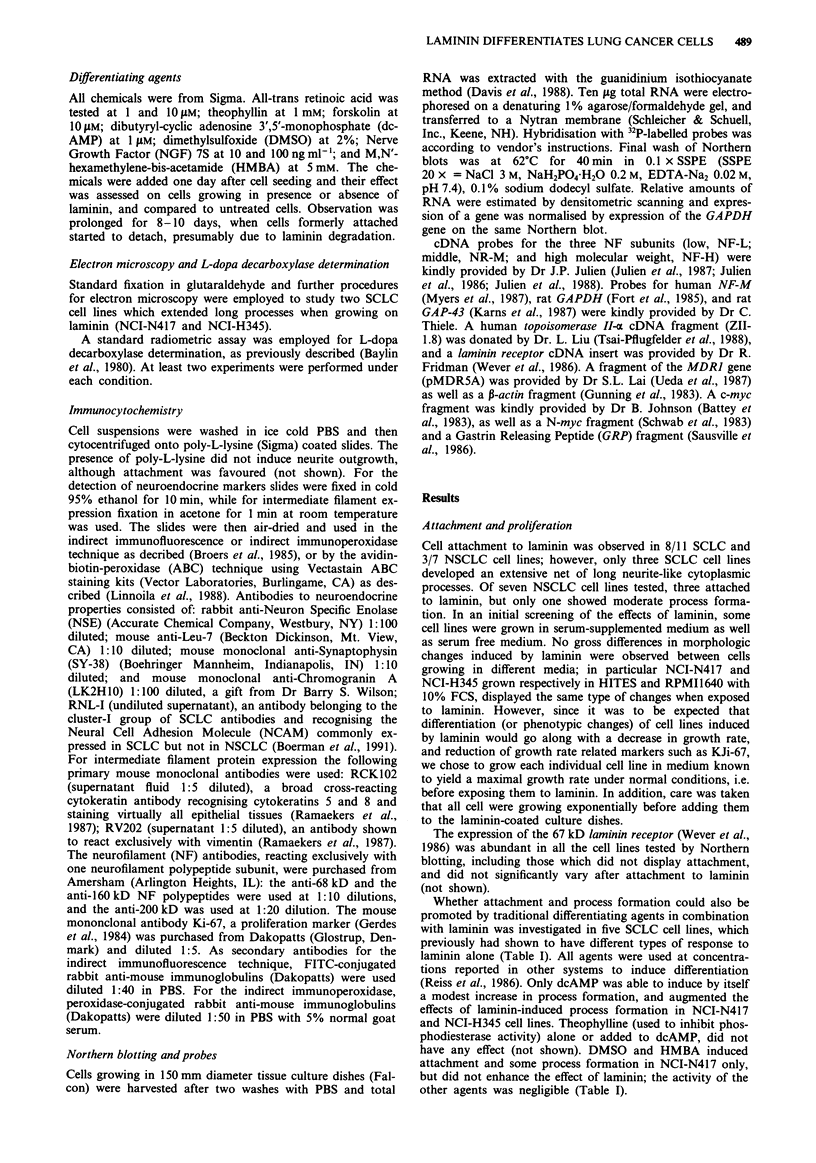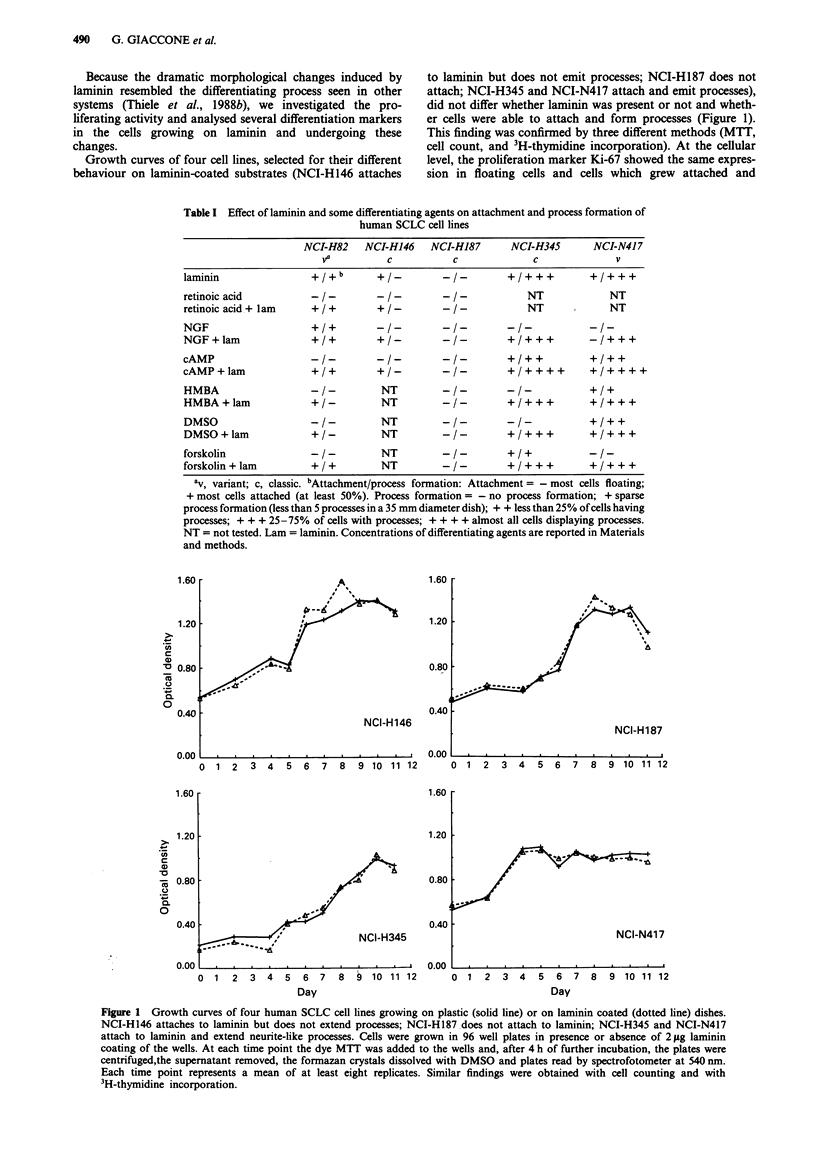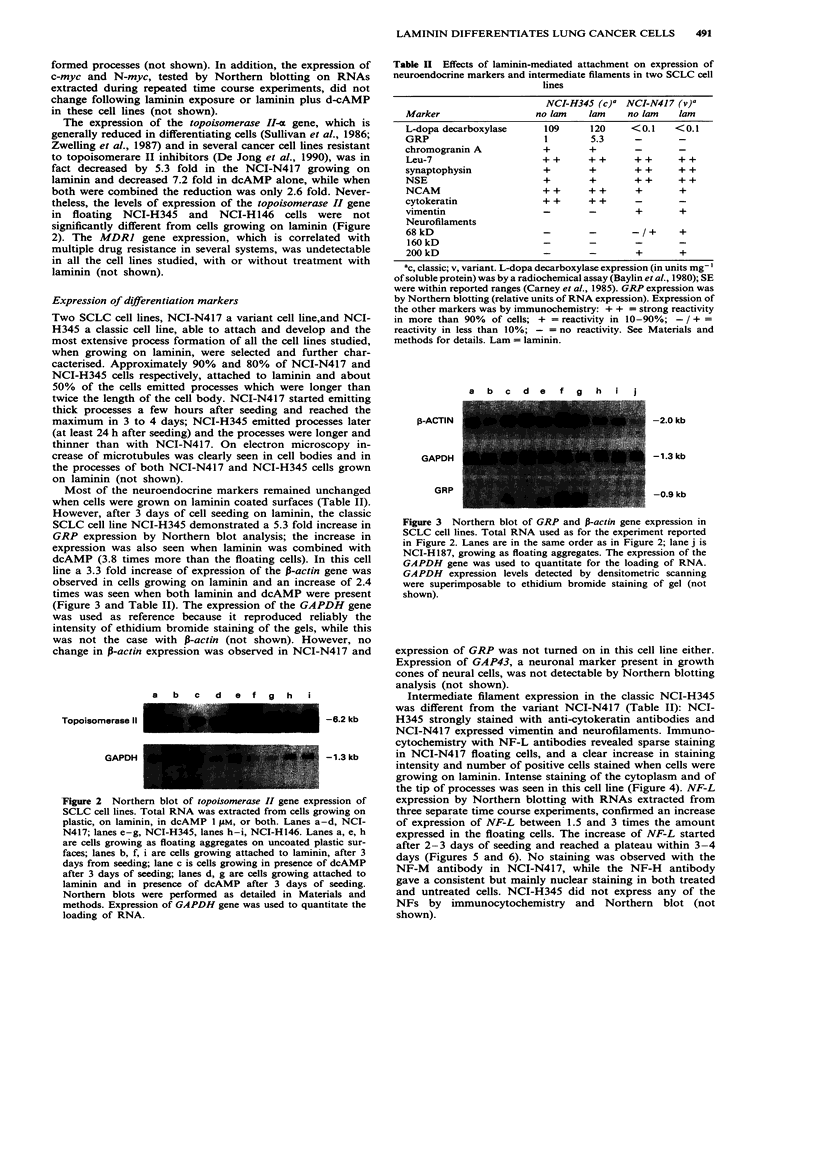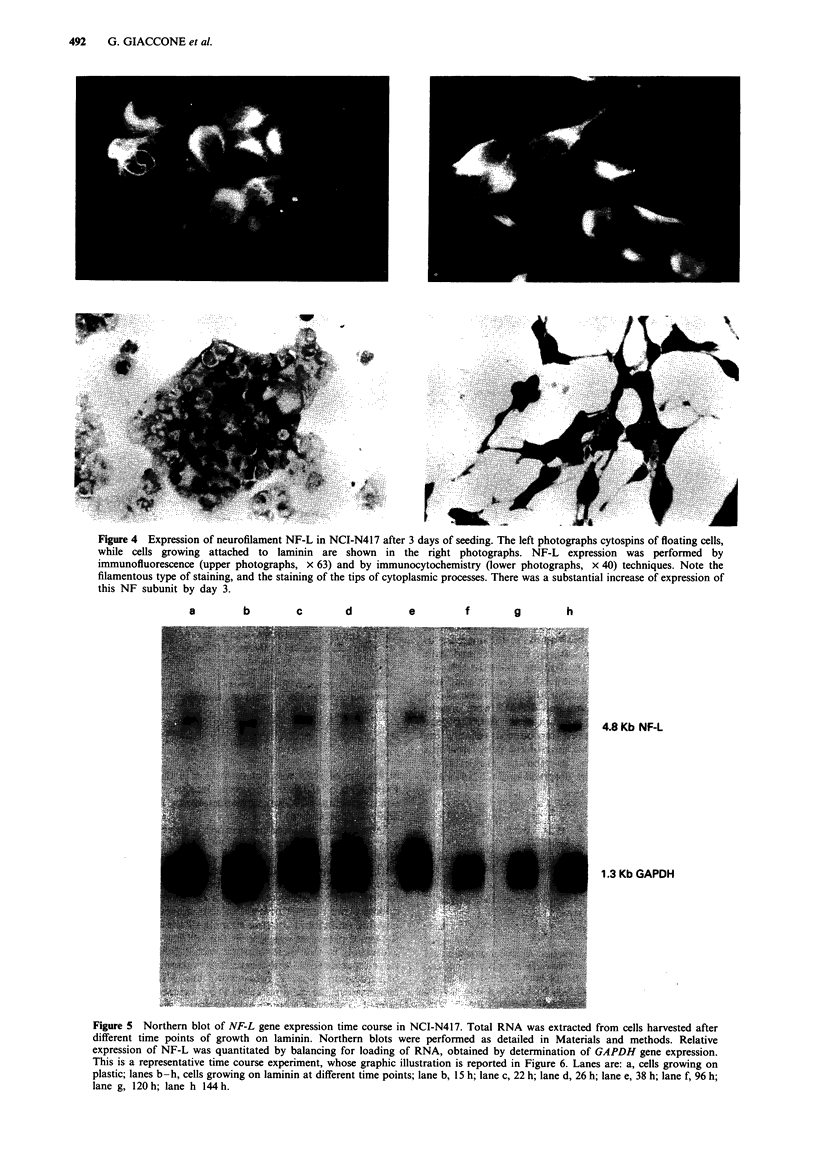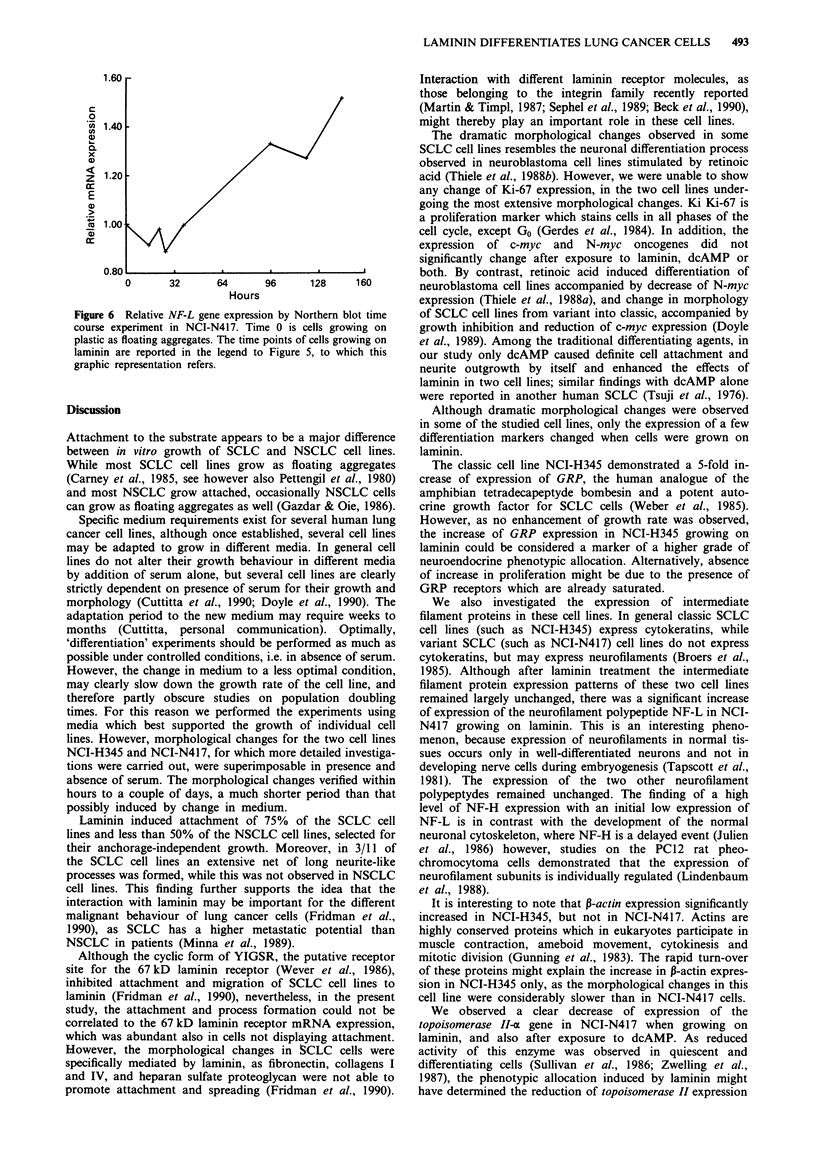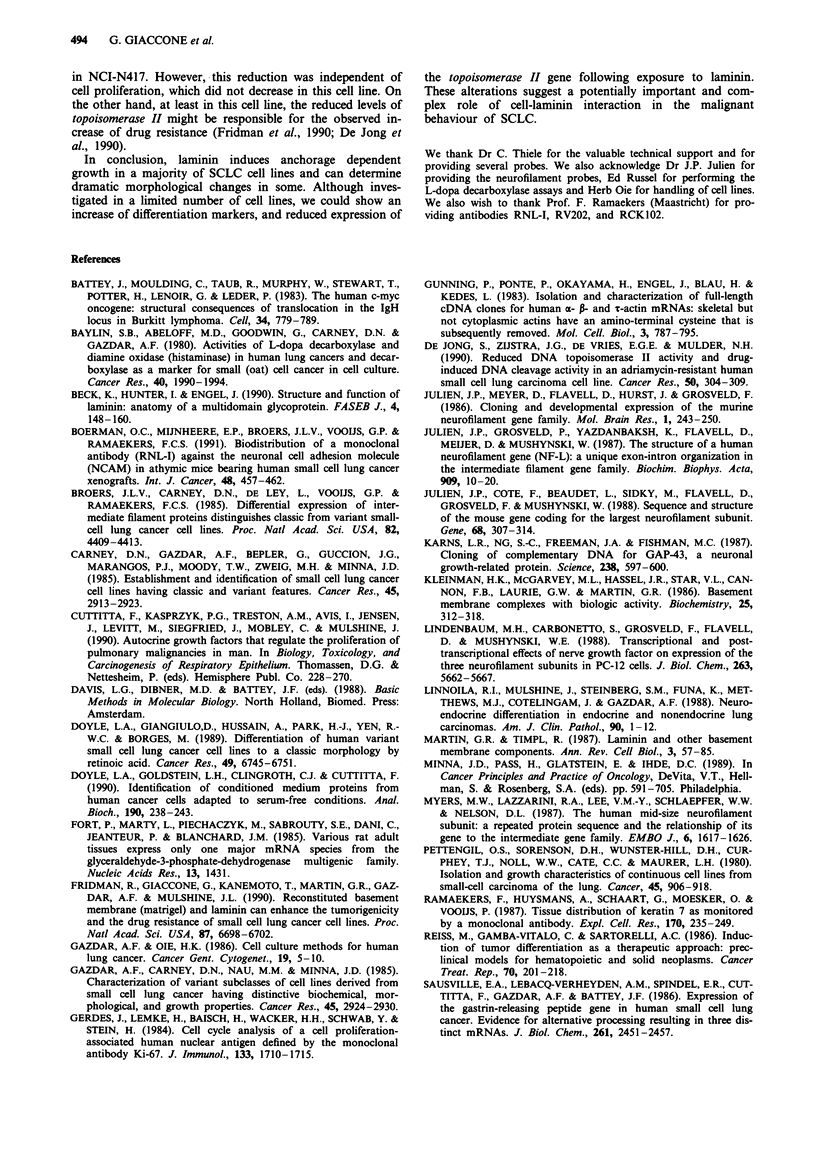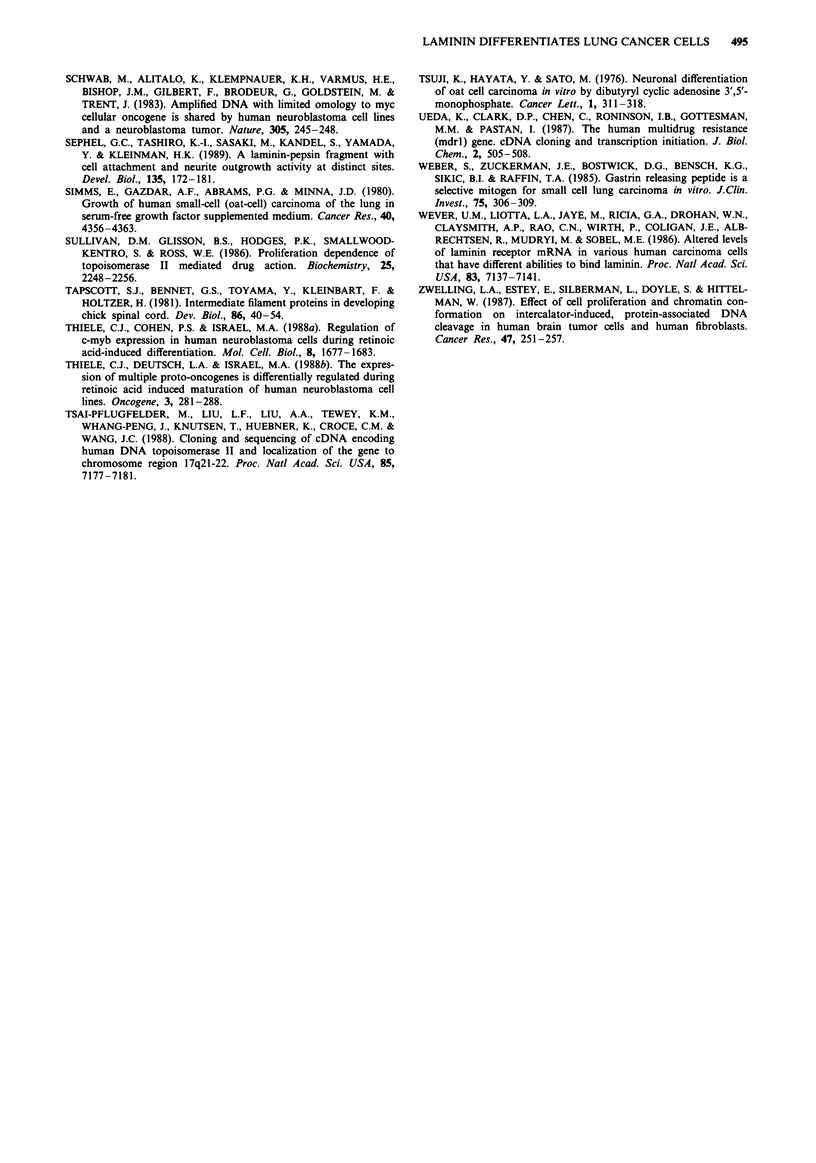# Increased expression of differentiation markers can accompany laminin-induced attachment of small cell lung cancer cells.

**DOI:** 10.1038/bjc.1992.301

**Published:** 1992-09

**Authors:** G. Giaccone, J. Broers, S. Jensen, R. I. Fridman, R. Linnoila, A. F. Gazdar

**Affiliations:** National Cancer Institute, NCI-Navy Medical Oncology Branch, National Institutes of Health, Bethesda, Maryland.

## Abstract

**Images:**


Br. J. Cancer (1992), 66, 488–495 © Macmillan Press Ltd., 1992

### Increased expression of differentiation markers can accompany laminin-induced attachment of small cell lung cancer cells

G. Giaccone[1]†, J. Broers[1]‡ S. Jensen[1], R.I. Fridman[2], R. Linnoila[1] & A.F. Gazdar[1]*

[1]*National Cancer Institute, NCI-Navy Medical Oncology Branch, National Institutes of Health, Bethesda, Maryland;* [2]*Laboratory of Developmental Biology and Anomalies, National Institute of Dental Research, National Institute of Health, Bethesda, Maryland, USA.*

**Summary** We investigated the interaction between human lung cancer cells, laminin, and several differentiating agents. When grown on laminin coated substrate eight out of 11 small cell lung cancer (SCLC) cell lines exhibited attachment to laminin and three had extensive outgrowth of long neurite-like processes. Of seven non-small cell lung cancer cell lines, selected for their *in vitro* anchorage-independent growth, attachment was observed in only three cell lines, and process formation was far less extensive than in SCLC cell lines. Among several differentiating agents, only dcAMP, which alone induced attachment and some process formation, increased laminin-mediated attachment and process formation of two SCLC cell lines, NCI-N417 a variant cell line, and NCI-H345, a classic cell line. The expression of several neuroendocrine and neuronal markers was investigated in these two SCLC cell lines. The expression of the light subunit of neurofilaments increased in NCI-N417 within 3 to 4 days of seeding, while NCI-H345 exhibited approximately 5 fold increase in expression of the *GRP* gene and a 3 fold increase expression of the *β-actin* gene. The expression of a number of other neuroendocrine and neuronal markers did not change following growth on laminin. The doubling times remained unchanged independent of the presence of and attachment to laminin while *topoisomerase II* gene expression levels in NCI-N417 cells decreased approximately 5 fold when cells were growing on laminin.

Among the four major histological types of lung cancer, small cell lung cancer (SCLC) is characterised by the development of early and widespread metastases and by initial sensitivity to chemotherapy. However, SCLC recurs in the majority of patients and becomes resistant to multiple antineoplastic drugs, causing the death of about 90% of patients in less than 2 years. The other three types of lung cancer, commonly referred to as non-small cell lung cancer (NSCLC), have a lower tendency to metastasise and are less sensitive to antineoplastic drugs than SCLC (Minna *et al.*, 1989).

Crossing basement membranes is an important step in metastasis formation. Tumour cell attachment to laminin, the major component of basement membranes, followed by production and release of proteolytic enzymes by the tumour cell (such as collagenase IV and plasminogen activator) is thought to play an important role in metastasis formation (Martin & Timpl, 1987). Moreover, binding to laminin elicits cell-specific responses in different cell types, like cell polarisation, differentiation, neurite outgrowth, migration, and cell growth (Martin & Timpl, 1987; Beck *et al.*, 1990).

Laminin can determine cell attachment of some SCLC cells, which usually grow in suspension, followed by dramatic morphological changes; the attachment to laminin was also shown to be associated with an increased resistance to several cytotoxic agents commonly used in the treatment of SCLC patients (Fridman *et al.*, 1990).

In this paper we describe the ability of human lung cancer cell lines to undergo attachment and process formation when grown on laminin coated surfaces, and in presence of traditional differentiating agents. We report in particular the characterisation of the changes taking place in certain SCLC cell lines, concerning expression of intermediate filament proteins, and several neuroendocrine and neuronal markers.

Correspondence: G. Giaccone, Free University Hospital, Department of Oncology, De Boelelaan 1117, 1081 HV, Amsterdam, The Netherlands.

Present addresses: *Department of Pathology, University of Texas Southwestern Medical Center, Dallas, TX. †Department of Oncology, Free University Hospital, Amsterdam, The Netherlands; ‡Department of Molecular and Cell Biology, University of Limburg, Maastricht, The Netherlands.

Received 17 December 1991; and in revised form 18 May 1992.

### Materials and methods

### *Cell lines and growth curves*

The human lung cancer cell lines used were established and characterised as described previously (Carney *et al.*, 1985; Gazdar *et al.*, 1985). They were maintained in a humidified incubator with 5% $CO_2$ and air, at 37°C. All the cell lines studied were selected because they were growing as floating aggregates, including seven NSCLC cell lines, which more frequently grow as adherent monolayers (Gazdar & Oie, 1986). NSCLC cell lines did not express neuroendocrine properties, in contrast to SCLC cell lines. Cell lines were maintained in the medium which best supported their growth; this was RPMI1640 with 10% FCS for all SCLC cell lines except NCI-H345 which grew better in HITES serum-free medium (Simms *et al.*, 1980). NSCLC cell lines were all grown in ACL-4 serum-free medium (Gazdar & Oie, 1986), except a NSCLC cell line which grew best in HITES plus 2.5% FCS. Cell lines were tested and found to be free of Mycoplasma contamination.

Proliferation and doubling times were assessed by cell counting, by MTT assay and by $^3$H-thymidine incorporation. The dye MTT (3[4,5-dimethylthiazol-2-yl]-2,5-diphenyl tetrazolium bromide) (Sigma Chemical Co., St. Louis, MO), stains only metabolically active cells and the number of cells is proportional to the intensity of staining. Thymidine incorporation provides an estimate of proliferating cells which incorporate thymidine during the S phase of the cell cycle.

### *Laminin*

Laminin, extracted as previously described (Kleinman *et al.*, 1986), was diluted in PBS, placed into the wells and incubated at 37°C for 1 h. The supernatant was then gently removed, replaced by 2% BSA, and incubated at 37°C for another hour, to block non-specific binding sites on the plastic surface. The use of BSA did not influence the results of the attachment experiments (not shown). Two washes with PBS followed, and cells were then seeded. Tissue culture dishes (35 mm diameter, Falcon, Lincoln Park, NJ) were coated with 10 μg of laminin, while 5 μg and 2 μg were placed into wells of 24 or 96 well plates (Costar, Cambridge, MA), respectively. Slides for immunocytochemistry were coated with approximately 30 μg of laminin.

*Differentiating agents*

All chemicals were from Sigma. All-trans retinoic acid was tested at 1 and 10 μM; theophyllin at 1 mM; forskolin at 10 μM; dibutyryl-cyclic adenosine 3',5'-monophosphate (dc-AMP) at 1 μM; dimethylsulfoxide (DMSO) at 2%; Nerve Growth Factor (NGF) 7S at 10 and 100 ng $ml^{-1}$; and M,N'-hexamethylene-bis-acetamide (HMBA) at 5 mM. The chemicals were added one day after cell seeding and their effect was assessed on cells growing in presence or absence of laminin, and compared to untreated cells. Observation was prolonged for 8–10 days, when cells formerly attached started to detach, presumably due to laminin degradation.

*Electron microscopy and L-dopa decarboxylase determination*

Standard fixation in glutaraldehyde and further procedures for electron microscopy were employed to study two SCLC cell lines which extended long processes when growing on laminin (NCI-N417 and NCI-H345).

A standard radiometric assay was employed for L-dopa decarboxylase determination, as previously described (Baylin *et al.*, 1980). At least two experiments were performed under each condition.

*Immunocytochemistry*

Cell suspensions were washed in ice cold PBS and then cytocentrifuged onto poly-L-lysine (Sigma) coated slides. The presence of poly-L-lysine did not induce neurite outgrowth, although attachment was favoured (not shown). For the detection of neuroendocrine markers slides were fixed in cold 95% ethanol for 10 min, while for intermediate filament expression fixation in acetone for 1 min at room temperature was used. The slides were then air-dried and used in the indirect immunofluorescence or indirect immunoperoxidase technique as decribed (Broers *et al.*, 1985), or by the avidin-biotin-peroxidase (ABC) technique using Vectastain ABC staining kits (Vector Laboratories, Burlingame, CA) as described (Linnoila *et al.*, 1988). Antibodies to neuroendocrine properties consisted of: rabbit anti-Neuron Specific Enolase (NSE) (Accurate Chemical Company, Westbury, NY) 1:100 diluted; mouse anti-Leu-7 (Beckton Dickinson, Mt. View, CA) 1:10 diluted; mouse monoclonal anti-Synaptophysin (SY-38) (Boehringer Mannheim, Indianapolis, IN) 1:10 diluted; and mouse monoclonal anti-Chromogranin A (LK2H10) 1:100 diluted, a gift from Dr Barry S. Wilson; RNL-I (undiluted supernatant), an antibody belonging to the cluster-I group of SCLC antibodies and recognising the Neural Cell Adhesion Molecule (NCAM) commonly expressed in SCLC but not in NSCLC (Boerman *et al.*, 1991). For intermediate filament protein expression the following primary mouse monoclonal antibodies were used: RCK102 (supernatant fluid 1:5 diluted), a broad cross-reacting cytokeratin antibody recognising cytokeratins 5 and 8 and staining virtually all epithelial tissues (Ramaekers *et al.*, 1987); RV202 (supernatant 1:5 diluted), an antibody shown to react exclusively with vimentin (Ramaekers *et al.*, 1987). The neurofilament (NF) antibodies, reacting exclusively with one neurofilament polypeptide subunit, were purchased from Amersham (Arlington Heights, IL): the anti-68 kD and the anti-160 kD NF polypeptides were used at 1:10 dilutions, and the anti-200 kD was used at 1:20 dilution. The mouse mononclonal antibody Ki-67, a proliferation marker (Gerdes *et al.*, 1984) was purchased from Dakopatts (Glostrup, Denmark) and diluted 1:5. As secondary antibodies for the indirect immunofluorescence technique, FITC-conjugated rabbit anti-mouse immunoglobulins (Dakopatts) were used diluted 1:40 in PBS. For the indirect immunoperoxidase, peroxidase-conjugated rabbit anti-mouse immunoglobulins (Dakopatts) were diluted 1:50 in PBS with 5% normal goat serum.

*Northern blotting and probes*

Cells growing in 150 mm diameter tissue culture dishes (Falcon) were harvested after two washes with PBS and total RNA was extracted with the guanidinium isothiocyanate method (Davis *et al.*, 1988). Ten μg total RNA were electrophoresed on a denaturing 1% agarose/formaldehyde gel, and transferred to a Nytran membrane (Schleicher & Schuell, Inc., Keene, NH). Hybridisation with $^{32}P$-labelled probes was according to vendor's instructions. Final wash of Northern blots was at 62°C for 40 min in 0.1 × SSPE (SSPE 20 × = NaCl 3 M, $NaH_2PO_4{\cdot}H_2O$ 0.2 M, EDTA-$Na_2$ 0.02 M, pH 7.4), 0.1% sodium dodecyl sulfate. Relative amounts of RNA were estimated by densitometric scanning and expression of a gene was normalised by expression of the *GAPDH* gene on the same Northern blot.

cDNA probes for the three NF subunits (low, NF-L; middle, NR-M; and high molecular weight, NF-H) were kindly provided by Dr J.P. Julien (Julien *et al.*, 1987; Julien *et al.*, 1986; Julien *et al.*, 1988). Probes for human *NF-M* (Myers *et al.*, 1987), rat *GAPDH* (Fort *et al.*, 1985), and rat *GAP-43* (Karns *et al.*, 1987) were kindly provided by Dr C. Thiele. A human *topoisomerase II-α* cDNA fragment (ZII-1.8) was donated by Dr. L. Liu (Tsai-Pflugfelder *et al.*, 1988), and a *laminin receptor* cDNA insert was provided by Dr R. Fridman (Wever *et al.*, 1986). A fragment of the *MDR1* gene (pMDR5A) was provided by Dr S.L. Lai (Ueda *et al.*, 1987) as well as a *β-actin* fragment (Gunning *et al.*, 1983). A c-*myc* fragment was kindly provided by Dr B. Johnson (Battey *et al.*, 1983), as well as a N-*myc* fragment (Schwab *et al.*, 1983) and a Gastrin Releasing Peptide (*GRP*) fragment (Sausville *et al.*, 1986).

### Results

*Attachment and proliferation*

Cell attachment to laminin was observed in 8/11 SCLC and 3/7 NSCLC cell lines; however, only three SCLC cell lines developed an extensive net of long neurite-like cytoplasmic processes. Of seven NSCLC cell lines tested, three attached to laminin, but only one showed moderate process formation. In an initial screening of the effects of laminin, some cell lines were grown in serum-supplemented medium as well as serum free medium. No gross differences in morphologic changes induced by laminin were observed between cells growing in different media; in particular NCI-N417 and NCI-H345 grown respectively in HITES and RPMI1640 with 10% FCS, displayed the same type of changes when exposed to laminin. However, since it was to be expected that differentiation (or phenotypic changes) of cell lines induced by laminin would go along with a decrease in growth rate, and reduction of growth rate related markers such as KJi-67, we chose to grow each individual cell line in medium known to yield a maximal growth rate under normal conditions, i.e. before exposing them to laminin. In addition, care was taken that all cell were growing exponentially before adding them to the laminin-coated culture dishes.

The expression of the 67 kD *laminin receptor* (Wever *et al.*, 1986) was abundant in all the cell lines tested by Northern blotting, including those which did not display attachment, and did not significantly vary after attachment to laminin (not shown).

Whether attachment and process formation could also be promoted by traditional differentiating agents in combination with laminin was investigated in five SCLC cell lines, which previously had shown to have different types of response to laminin alone (Table I). All agents were used at concentrations reported in other systems to induce differentiation (Reiss *et al.*, 1986). Only dcAMP was able to induce by itself a modest increase in process formation, and augmented the effects of laminin-induced process formation in NCI-N417 and NCI-H345 cell lines. Theophylline (used to inhibit phosphodiesterase activity) alone or added to dcAMP, did not have any effect (not shown). DMSO and HMBA induced attachment and some process formation in NCI-N417 only, but did not enhance the effect of laminin; the activity of the other agents was negligible (Table I).

Because the dramatic morphological changes induced by laminin resembled the differentiating process seen in other systems (Thiele *et al.*, 1988*b*), we investigated the proliferating activity and analysed several differentiation markers in the cells growing on laminin and undergoing these changes.

Growth curves of four cell lines, selected for their different behaviour on laminin-coated substrates (NCI-H146 attaches to laminin but does not emit processes; NCI-H187 does not attach; NCI-H345 and NCI-N417 attach and emit processes), did not differ whether laminin was present or not and whether cells were able to attach and form processes (Figure 1). This finding was confirmed by three different methods (MTT, cell count, and $^3$H-thymidine incorporation). At the cellular level, the proliferation marker Ki-67 showed the same expression in floating cells and cells which grew attached and

**Table I** Effect of laminin and some differentiating agents on attachment and process formation of human SCLC cell lines

| | *NCI-H82* *v*[a] | *NCI-H146* *c* | *NCI-H187* *c* | *NCI-H345* *c* | *NCI-N417* *v* |
|---|---|---|---|---|---|
| laminin | +/+[b] | +/− | −/− | +/+++ | +/+++ |
| retinoic acid | −/− | −/− | −/− | NT | NT |
| retinoic acid + lam | +/+ | +/− | −/− | NT | NT |
| NGF | +/+ | −/− | −/− | −/− | −/− |
| NGF + lam | +/+ | +/− | −/− | +/+++ | −/+++ |
| cAMP | −/− | −/− | −/− | +/++ | +/++ |
| cAMP + lam | +/+ | +/− | −/− | +/++++ | +/++++ |
| HMBA | −/− | NT | −/− | −/− | +/+ |
| HMBA + lam | +/− | NT | −/− | +/+++ | +/+++ |
| DMSO | −/− | NT | −/− | −/− | +/++ |
| DMSO + lam | +/− | NT | −/− | +/+++ | +/+++ |
| forskolin | −/− | NT | −/− | +/+ | −/− |
| forskolin + lam | +/+ | NT | −/− | +/+++ | +/+++ |

[a]v, variant; c, classic. [b]Attachment/process formation: Attachment = − most cells floating; + most cells attached (at least 50%). Process formation = − no process formation; + sparse process formation (less than 5 processes in a 35 mm diameter dish); + + less than 25% of cells having processes; + + + 25–75% of cells with processes; + + + + almost all cells displaying processes. NT = not tested. Lam = laminin. Concentrations of differentiating agents are reported in Materials and methods.

**Figure 1** Growth curves of four human SCLC cell lines growing on plastic (solid line) or on laminin coated (dotted line) dishes. NCI-H146 attaches to laminin but does not extend processes; NCI-H187 does not attach to laminin; NCI-H345 and NCI-N417 attach to laminin and extend neurite-like processes. Cells were grown in 96 well plates in presence or absence of 2 µg laminin coating of the wells. At each time point the dye MTT was added to the wells and, after 4 h of further incubation, the plates were centrifuged,the supernatant removed, the formazan crystals dissolved with DMSO and plates read by spectrofotometer at 540 nm. Each time point represents a mean of at least eight replicates. Similar findings were obtained with cell counting and with $^3$H-thymidine incorporation.

formed processes (not shown). In addition, the expression of c-*myc* and N-*myc*, tested by Northern blotting on RNAs extracted during repeated time course experiments, did not change following laminin exposure or laminin plus d-cAMP in these cell lines (not shown).

The expression of the *topoisomerase II-α* gene, which is generally reduced in differentiating cells (Sullivan *et al.*, 1986; Zwelling *et al.*, 1987) and in several cancer cell lines resistant to topoisomerare II inhibitors (De Jong *et al.*, 1990), was in fact decreased by 5.3 fold in the NCI-N417 growing on laminin and decreased 7.2 fold in dcAMP alone, while when both were combined the reduction was only 2.6 fold. Nevertheless, the levels of expression of the *topoisomerase II* gene in floating NCI-H345 and NCI-H146 cells were not significantly different from cells growing on laminin (Figure 2). The *MDR1* gene expression, which is correlated with multiple drug resistance in several systems, was undetectable in all the cell lines studied, with or without treatment with laminin (not shown).

*Expression of differentiation markers*

Two SCLC cell lines, NCI-N417 a variant cell line,and NCI-H345 a classic cell line, able to attach and develop and the most extensive process formation of all the cell lines studied, when growing on laminin, were selected and further charcacterised. Approximately 90% and 80% of NCI-N417 and NCI-H345 cells respectively, attached to laminin and about 50% of the cells emitted processes which were longer than twice the length of the cell body. NCI-N417 started emitting thick processes a few hours after seeding and reached the maximum in 3 to 4 days; NCI-H345 emitted processes later (at least 24 h after seeding) and the processes were longer and thinner than with NCI-N417. On electron microscopy increase of microtubules was clearly seen in cell bodies and in the processes of both NCI-N417 and NCI-H345 cells grown on laminin (not shown).

Most of the neuroendocrine markers remained unchanged when cells were grown on laminin coated surfaces (Table II). However, after 3 days of cell seeding on laminin, the classic SCLC cell line NCI-H345 demonstrated a 5.3 fold increase in *GRP* expression by Northern blot analysis; the increase in expression was also seen when laminin was combined with dcAMP (3.8 times more than the floating cells). In this cell line a 3.3 fold increase of expression of the *β-actin* gene was observed in cells growing on laminin and an increase of 2.4 times was seen when both laminin and dcAMP were present (Figure 3 and Table II). The expression of the *GAPDH* gene was used as reference because it reproduced reliably the intensity of ethidium bromide staining of the gels, while this was not the case with *β-actin* (not shown). However, no change in *β-actin* expression was observed in NCI-N417 and expression of *GRP* was not turned on in this cell line either. Expression of *GAP43*, a neuronal marker present in growth cones of neural cells, was not detectable by Northern blotting analysis (not shown).

Intermediate filament expression in the classic NCI-H345 was different from the variant NCI-N417 (Table II): NCI-H345 strongly stained with anti-cytokeratin antibodies and NCI-N417 expressed vimentin and neurofilaments. Immunocytochemistry with NF-L antibodies revealed sparse staining in NCI-N417 floating cells, and a clear increase in staining intensity and number of positive cells stained when cells were growing on laminin. Intense staining of the cytoplasm and of the tip of processes was seen in this cell line (Figure 4). *NF-L* expression by Northern blotting with RNAs extracted from three separate time course experiments, confirmed an increase of expression of *NF-L* between 1.5 and 3 times the amount expressed in the floating cells. The increase of *NF-L* started after 2–3 days of seeding and reached a plateau within 3–4 days (Figures 5 and 6). No staining was observed with the NF-M antibody in NCI-N417, while the NF-H antibody gave a consistent but mainly nuclear staining in both treated and untreated cells. NCI-H345 did not express any of the NFs by immunocytochemistry and Northern blot (not shown).

**Table II** Effects of laminin-mediated attachment on expression of neuroendocrine markers and intermediate filaments in two SCLC cell lines

| | *NCI-H345 (c)*[a] | | *NCI-N417 (v)*[a] | |
|---|---|---|---|---|
| *Marker* | *no lam* | *lam* | *no lam* | *lam* |
| L-dopa decarboxylase | 109 | 120 | <0.1 | <0.1 |
| GRP | 1 | 5.3 | – | – |
| chromogranin A | + | + | – | – |
| Leu-7 | ++ | ++ | ++ | ++ |
| synaptophysin | + | + | ++ | ++ |
| NSE | + | + | ++ | ++ |
| NCAM | ++ | ++ | + | + |
| cytokeratin | ++ | ++ | – | – |
| vimentin | – | – | + | + |
| Neurofilaments | | | | |
| 68 kD | – | – | –/+ | + |
| 160 kD | – | – | – | – |
| 200 kD | – | – | + | + |

[a]c, classic; v, variant. L-dopa decarboxylase expression (in units $mg^{-1}$ of soluble protein) was by a radiochemical assay (Baylin *et al.*, 1980); SE were within reported ranges (Carney *et al.*, 1985). *GRP* expression was by Northern blotting (relative units of RNA expression). Expression of the other markers was by immunochemistry: ++ = strong reactivity in more than 90% of cells; + = reactivity in 10–90%; –/+ = reactivity in less than 10%; – = no reactivity. See Materials and methods for details. Lam = laminin.

**Figure 3** Northern blot of *GRP* and *β-actin* gene expression in SCLC cell lines. Total RNA used as for the experiment reported in Figure 2. Lanes are in the same order as in Figure 2; lane j is NCI-H187, growing as floating aggregates. The expression of the *GAPDH* gene was used to quantitate for the loading of RNA. *GAPDH* expression levels detected by densitometric scanning were superimposable to ethidium bromide staining of gel (not shown).

**Figure 2** Northern blot of *topoisomerase II* gene expression of SCLC cell lines. Total RNA was extracted from cells growing on plastic, on laminin, in dcAMP 1 μM, or both. Lanes a–d, NCI-N417; lanes e–g, NCI-H345, lanes h–i, NCI-H146. Lanes a, e, h are cells growing as floating aggregates on uncoated plastic surfaces; lanes b, f, i are cells growing attached to laminin, after 3 days from seeding; lane c is cells growing in presence of dcAMP after 3 days of seeding; lanes d, g are cells growing attached to laminin and in presence of dcAMP after 3 days of seeding. Northern blots were performed as detailed in Materials and methods. Expression of *GAPDH* gene was used to quantitate the loading of RNA.

**Figure 4** Expression of neurofilament NF-L in NCI-N417 after 3 days of seeding. The left photographs cytospins of floating cells, while cells growing attached to laminin are shown in the right photographs. NF-L expression was performed by immunofluorescence (upper photographs, × 63) and by immunocytochemistry (lower photographs, × 40) techniques. Note the filamentous type of staining, and the staining of the tips of cytoplasmic processes. There was a substantial increase of expression of this NF subunit by day 3.

**Figure 5** Northern blot of *NF-L* gene expression time course in NCI-N417. Total RNA was extracted from cells harvested after different time points of growth on laminin. Northern blots were performed as detailed in Materials and methods. Relative expression of NF-L was quantitated by balancing for loading of RNA, obtained by determination of *GAPDH* gene expression. This is a representative time course experiment, whose graphic illustration is reported in Figure 6. Lanes are: a, cells growing on plastic; lanes b–h, cells growing on laminin at different time points; lane b, 15 h; lane c, 22 h; lane d, 26 h; lane e, 38 h; lane f, 96 h; lane g, 120 h; lane h 144 h.

**Figure 6** Relative *NF-L* gene expression by Northern blot time course experiment in NCI-N417. Time 0 is cells growing on plastic as floating aggregates. The time points of cells growing on laminin are reported in the legend to Figure 5, to which this graphic representation refers.

### Discussion

Attachment to the substrate appears to be a major difference between *in vitro* growth of SCLC and NSCLC cell lines. While most SCLC cell lines grow as floating aggregates (Carney *et al.*, 1985, see however also Pettengil *et al.*, 1980) and most NSCLC grow attached, occasionally NSCLC cells can grow as floating aggregates as well (Gazdar & Oie, 1986).

Specific medium requirements exist for several human lung cancer cell lines, although once established, several cell lines may be adapted to grow in different media. In general cell lines do not alter their growth behaviour in different media by addition of serum alone, but several cell lines are clearly strictly dependent on presence of serum for their growth and morphology (Cuttitta *et al.*, 1990; Doyle *et al.*, 1990). The adaptation period to the new medium may require weeks to months (Cuttitta, personal communication). Optimally, 'differentiation' experiments should be performed as much as possible under controlled conditions, i.e. in absence of serum. However, the change in medium to a less optimal condition, may clearly slow down the growth rate of the cell line, and therefore partly obscure studies on population doubling times. For this reason we performed the experiments using media which best supported the growth of individual cell lines. However, morphological changes for the two cell lines NCI-H345 and NCI-N417, for which more detailed investigations were carried out, were superimposable in presence and absence of serum. The morphological changes verified within hours to a couple of days, a much shorter period than that possibly induced by change in medium.

Laminin induced attachment of 75% of the SCLC cell lines and less than 50% of the NSCLC cell lines, selected for their anchorage-independent growth. Moreover, in 3/11 of the SCLC cell lines an extensive net of long neurite-like processes was formed, while this was not observed in NSCLC cell lines. This finding further supports the idea that the interaction with laminin may be important for the different malignant behaviour of lung cancer cells (Fridman *et al.*, 1990), as SCLC has a higher metastatic potential than NSCLC in patients (Minna *et al.*, 1989).

Although the cyclic form of YIGSR, the putative receptor site for the 67 kD laminin receptor (Wever *et al.*, 1986), inhibited attachment and migration of SCLC cell lines to laminin (Fridman *et al.*, 1990), nevertheless, in the present study, the attachment and process formation could not be correlated to the 67 kD laminin receptor mRNA expression, which was abundant also in cells not displaying attachment. However, the morphological changes in SCLC cells were specifically mediated by laminin, as fibronectin, collagens I and IV, and heparan sulfate proteoglycan were not able to promote attachment and spreading (Fridman *et al.*, 1990). Interaction with different laminin receptor molecules, as those belonging to the integrin family recently reported (Martin & Timpl, 1987; Sephel *et al.*, 1989; Beck *et al.*, 1990), might thereby play an important role in these cell lines.

The dramatic morphological changes observed in some SCLC cell lines resembles the neuronal differentiation process observed in neuroblastoma cell lines stimulated by retinoic acid (Thiele *et al.*, 1988*b*). However, we were unable to show any change of Ki-67 expression, in the two cell lines undergoing the most extensive morphological changes. Ki Ki-67 is a proliferation marker which stains cells in all phases of the cell cycle, except $G_0$ (Gerdes *et al.*, 1984). In addition, the expression of c-*myc* and N-*myc* oncogenes did not significantly change after exposure to laminin, dcAMP or both. By contrast, retinoic acid induced differentiation of neuroblastoma cell lines accompanied by decrease of N-*myc* expression (Thiele *et al.*, 1988*a*), and change in morphology of SCLC cell lines from variant into classic, accompanied by growth inhibition and reduction of c-*myc* expression (Doyle *et al.*, 1989). Among the traditional differentiating agents, in our study only dcAMP caused definite cell attachment and neurite outgrowth by itself and enhanced the effects of laminin in two cell lines; similar findings with dcAMP alone were reported in another human SCLC (Tsuji *et al.*, 1976).

Although dramatic morphological changes were observed in some of the studied cell lines, only the expression of a few differentiation markers changed when cells were grown on laminin.

The classic cell line NCI-H345 demonstrated a 5-fold increase of expression of *GRP*, the human analogue of the amphibian tetradecapeptyde bombesin and a potent autocrine growth factor for SCLC cells (Weber *et al.*, 1985). However, as no enhancement of growth rate was observed, the increase of *GRP* expression in NCI-H345 growing on laminin could be considered a marker of a higher grade of neuroendocrine phenotypic allocation. Alternatively, absence of increase in proliferation might be due to the presence of GRP receptors which are already saturated.

We also investigated the expression of intermediate filament proteins in these cell lines. In general classic SCLC cell lines (such as NCI-H345) express cytokeratins, while variant SCLC (such as NCI-N417) cell lines do not express cytokeratins, but may express neurofilaments (Broers *et al.*, 1985). Although after laminin treatment the intermediate filament protein expression patterns of these two cell lines remained largely unchanged, there was a significant increase of expression of the neurofilament polypeptide NF-L in NCI-N417 growing on laminin. This is an interesting phenomenon, because expression of neurofilaments in normal tissues occurs only in well-differentiated neurons and not in developing nerve cells during embryogenesis (Tapscott *et al.*, 1981). The expression of the two other neurofilament polypeptydes remained unchanged. The finding of a high level of NF-H expression with an initial low expression of NF-L is in contrast with the development of the normal neuronal cytoskeleton, where NF-H is a delayed event (Julien *et al.*, 1986) however, studies on the PC12 rat pheochromocytoma cells demonstrated that the expression of neurofilament subunits is individually regulated (Lindenbaum *et al.*, 1988).

It is interesting to note that *β-actin* expression significantly increased in NCI-H345, but not in NCI-N417. Actins are highly conserved proteins which in eukaryotes participate in muscle contraction, ameboid movement, cytokinesis and mitotic division (Gunning *et al.*, 1983). The rapid turn-over of these proteins might explain the increase in *β*-actin expression in NCI-H345 only, as the morphological changes in this cell line were considerably slower than in NCI-N417 cells.

We observed a clear decrease of expression of the *topoisomerase II-α* gene in NCI-N417 when growing on laminin, and also after exposure to dcAMP. As reduced activity of this enzyme was observed in quiescent and differentiating cells (Sullivan *et al.*, 1986; Zwelling *et al.*, 1987), the phenotypic allocation induced by laminin might have determined the reduction of *topoisomerase II* expression in NCI-N417. However, this reduction was independent of cell proliferation, which did not decrease in this cell line. On the other hand, at least in this cell line, the reduced levels of *topoisomerase II* might be responsible for the observed increase of drug resistance (Fridman *et al.*, 1990; De Jong *et al.*, 1990).

In conclusion, laminin induces anchorage dependent growth in a majority of SCLC cell lines and can determine dramatic morphological changes in some. Although investigated in a limited number of cell lines, we could show an increase of differentiation markers, and reduced expression of the *topoisomerase II* gene following exposure to laminin. These alterations suggest a potentially important and complex role of cell-laminin interaction in the malignant behaviour of SCLC.

We thank Dr C. Thiele for the valuable technical support and for providing several probes. We also acknowledge Dr J.P. Julien for providing the neurofilament probes, Ed Russel for performing the L-dopa decarboxylase assays and Herb Oie for handling of cell lines. We also wish to thank Prof. F. Ramaekers (Maastricht) for providing antibodies RNL-I, RV202, and RCK102.

### References

BATTEY, J., MOULDING, C., TAUB, R., MURPHY, W., STEWART, T., POTTER, H., LENOIR, G. & LEDER, P. (1983). The human c-myc oncogene: structural consequences of translocation in the IgH locus in Burkitt lymphoma. *Cell*, **34**, 779-789.

BAYLIN, S.B., ABELOFF, M.D., GOODWIN, G., CARNEY, D.N. & GAZDAR, A.F. (1980). Activities of L-dopa decarboxylase and diamine oxidase (histaminase) in human lung cancers and decarboxylase as a marker for small (oat) cell cancer in cell culture. *Cancer Res.*, **40**, 1990-1994.

BECK, K., HUNTER, I. & ENGEL, J. (1990). Structure and function of laminin: anatomy of a multidomain glycoprotein. *FASEB J.*, **4**, 148-160.

BOERMAN, O.C., MIJNHEERE, E.P., BROERS, J.L.V., VOOIJS, G.P. & RAMAEKERS, F.C.S. (1991). Biodistribution of a monoclonal antibody (RNL-I) against the neuronal cell adhesion molecule (NCAM) in athymic mice bearing human small cell lung cancer xenografts. *Int. J. Cancer*, **48**, 457-462.

BROERS, J.L.V., CARNEY, D.N., DE LEY, L., VOOIJS, G.P. & RAMAEKERS, F.C.S. (1985). Differential expression of intermediate filament proteins distinguishes classic from variant small-cell lung cancer cell lines. *Proc. Natl Acad. Sci. USA*, **82**, 4409-4413.

CARNEY, D.N., GAZDAR, A.F., BEPLER, G., GUCCION, J.G., MARANGOS, P.J., MOODY, T.W., ZWEIG, M.H. & MINNA, J.D. (1985). Establishment and identification of small cell lung cancer cell lines having classic and variant features. *Cancer Res.*, **45**, 2913-2923.

CUTTITTA, F., KASPRZYK, P.G., TRESTON, A.M., AVIS, I., JENSEN, J., LEVITT, M., SIEGFRIED, J., MOBLEY, C. & MULSHINE, J. (1990). Autocrine growth factors that regulate the proliferation of pulmonary malignancies in man. In *Biology, Toxicology, and Carcinogenesis of Respiratory Epithelium*. Thomassen, D.G. & Nettesheim, P. (eds). Hemisphere Publ. Co. 228-270.

DAVIS, L.G., DIBNER, M.D. & BATTEY, J.F. (eds). (1988). *Basic Methods in Molecular Biology*. North Holland, Biomed. Press: Amsterdam.

DOYLE, L.A., GIANGIULO,D., HUSSAIN, A., PARK, H.-J., YEN, R.-W.C. & BORGES, M. (1989). Differentiation of human variant small cell lung cancer cell lines to a classic morphology by retinoic acid. *Cancer Res.*, **49**, 6745-6751.

DOYLE, L.A., GOLDSTEIN, L.H., CLINGROTH, C.J. & CUTTITTA, F. (1990). Identification of conditioned medium proteins from human cancer cells adapted to serum-free conditions. *Anal. Bioch.*, **190**, 238-243.

FORT, P., MARTY, L., PIECHACZYK, M., SABROUTY, S.E., DANI, C., JEANTEUR, P. & BLANCHARD, J.M. (1985). Various rat adult tissues express only one major mRNA species from the glyceraldehyde-3-phosphate-dehydrogenase multigenic family. *Nucleic Acids Res.*, **13**, 1431.

FRIDMAN, R., GIACCONE, G., KANEMOTO, T., MARTIN, G.R., GAZDAR, A.F. & MULSHINE, J.L. (1990). Reconstituted basement membrane (matrigel) and laminin can enhance the tumorigenicity and the drug resistance of small cell lung cancer cell lines. *Proc. Natl Acad. Sci. USA*, **87**, 6698-6702.

GAZDAR, A.F. & OIE, H.K. (1986). Cell culture methods for human lung cancer. *Cancer Gent. Cytogenet.*, **19**, 5-10.

GAZDAR, A.F., CARNEY, D.N., NAU, M.M. & MINNA, J.D. (1985). Characterization of variant subclasses of cell lines derived from small cell lung cancer having distinctive biochemical, morphological, and growth properties. *Cancer Res.*, **45**, 2924-2930.

GERDES, J., LEMKE, H., BAISCH, H., WACKER, H.H., SCHWAB, Y. & STEIN, H. (1984). Cell cycle analysis of a cell proliferation-associated human nuclear antigen defined by the monoclonal antibody Ki-67. *J. Immunol.*, **133**, 1710-1715.

GUNNING, P., PONTE, P., OKAYAMA, H., ENGEL, J., BLAU, H. & KEDES, L. (1983). Isolation and characterization of full-length cDNA clones for human α- β- and τ-actin mRNAs: skeletal but not cytoplasmic actins have an amino-terminal cysteine that is subsequently removed. *Mol. Cell. Biol.*, **3**, 787-795.

DE JONG, S., ZIJSTRA, J.G., DE VRIES, E.G.E. & MULDER, N.H. (1990). Reduced DNA topoisomerase II activity and drug-induced DNA cleavage activity in an adriamycin-resistant human small cell lung carcinoma cell line. *Cancer Res.*, **50**, 304-309.

JULIEN, J.P., MEYER, D., FLAVELL, D., HURST, J. & GROSVELD, F. (1986). Cloning and developmental expression of the murine neurofilament gene family. *Mol. Brain Res.*, **1**, 243-250.

JULIEN, J.P., GROSVELD, P., YAZDANBAKSH, K., FLAVELL, D., MEIJER, D. & MUSHYNSKI, W. (1987). The structure of a human neurofilament gene (NF-L): a unique exon-intron organization in the intermediate filament gene family. *Biochim. Biophys. Acta*, **909**, 10-20.

JULIEN, J.P., COTE, F., BEAUDET, L., SIDKY, M., FLAVELL, D., GROSVELD, F. & MUSHYNSKI, W. (1988). Sequence and structure of the mouse gene coding for the largest neurofilament subunit. *Gene*, **68**, 307-314.

KARNS, L.R., NG, S.-C., FREEMAN, J.A. & FISHMAN, M.C. (1987). Cloning of complementary DNA for GAP-43, a neuronal growth-related protein. *Science*, **238**, 597-600.

KLEINMAN, H.K., MCGARVEY, M.L., HASSEL, J.R., STAR, V.L., CANNON, F.B., LAURIE, G.W. & MARTIN, G.R. (1986). Basement membrane complexes with biologic activity. *Biochemistry*, **25**, 312-318.

LINDENBAUM, M.H., CARBONETTO, S., GROSVELD, F., FLAVELL, D. & MUSHYNSKI, W.E. (1988). Transcriptional and post-transcriptional effects of nerve growth factor on expression of the three neurofilament subunits in PC-12 cells. *J. Biol. Chem.*, **263**, 5662-5667.

LINNOILA, R.I., MULSHINE, J., STEINBERG, S.M., FUNA, K., METTHEWS, M.J., COTELINGAM, J. & GAZDAR, A.F. (1988). Neuroendocrine differentiation in endocrine and nonendocrine lung carcinomas. *Am. J. Clin. Pathol.*, **90**, 1-12.

MARTIN, G.R. & TIMPL, R. (1987). Laminin and other basement membrane components. *Ann. Rev. Cell Biol.*, **3**, 57-85.

MINNA, J.D., PASS, H., GLATSTEIN, E. & IHDE, D.C. (1989). In *Cancer Principles and Practice of Oncology*, DeVita, V.T., Hellman, S. & Rosenberg, S.A. (eds). pp. 591-705. Philadelphia.

MYERS, M.W., LAZZARINI, R.A., LEE, V.M.-Y., SCHLAEPFER, W.W. & NELSON, D.L. (1987). The human mid-size neurofilament subunit: a repeated protein sequence and the relationship of its gene to the intermediate gene family. *EMBO J.*, **6**, 1617-1626.

PETTENGIL, O.S., SORENSON, D.H., WUNSTER-HILL, D.H., CURPHEY, T.J., NOLL, W.W., CATE, C.C. & MAURER, L.H. (1980). Isolation and growth characteristics of continuous cell lines from small-cell carcinoma of the lung. *Cancer*, **45**, 906-918.

RAMAEKERS, F., HUYSMANS, A., SCHAART, G., MOESKER, O. & VOOIJS, P. (1987). Tissue distribution of keratin 7 as monitored by a monoclonal antibody. *Expl. Cell. Res.*, **170**, 235-249.

REISS, M., GAMBA-VITALO, C. & SARTORELLI, A.C. (1986). Induction of tumor differentiation as a therapeutic approach: preclinical models for hematopoietic and solid neoplasms. *Cancer Treat. Rep.*, **70**, 201-218.

SAUSVILLE, E.A., LEBACQ-VERHEYDEN, A.M., SPINDEL, E.R., CUTTITTA, F., GAZDAR, A.F. & BATTEY, J.F. (1986). Expression of the gastrin-releasing peptide gene in human small cell lung cancer. Evidence for alternative processing resulting in three distinct mRNAs. *J. Biol. Chem.*, **261**, 2451-2457.

SCHWAB, M., ALITALO, K., KLEMPNAUER, K.H., VARMUS, H.E., BISHOP, J.M., GILBERT, F., BRODEUR, G., GOLDSTEIN, M. & TRENT, J. (1983). Amplified DNA with limited omology to myc cellular oncogene is shared by human neuroblastoma cell lines and a neuroblastoma tumor. *Nature*, **305**, 245–248.

SEPHEL, G.C., TASHIRO, K.-I., SASAKI, M., KANDEL, S., YAMADA, Y. & KLEINMAN, H.K. (1989). A laminin-pepsin fragment with cell attachment and neurite outgrowth activity at distinct sites. *Devel. Biol.*, **135**, 172–181.

SIMMS, E., GAZDAR, A.F., ABRAMS, P.G. & MINNA, J.D. (1980). Growth of human small-cell (oat-cell) carcinoma of the lung in serum-free growth factor supplemented medium. *Cancer Res.*, **40**, 4356–4363.

SULLIVAN, D.M. GLISSON, B.S., HODGES, P.K., SMALLWOOD-KENTRO, S. & ROSS, W.E. (1986). Proliferation dependence of topoisomerase II mediated drug action. *Biochemistry*, **25**, 2248–2256.

TAPSCOTT, S.J., BENNET, G.S., TOYAMA, Y., KLEINBART, F. & HOLTZER, H. (1981). Intermediate filament proteins in developing chick spinal cord. *Dev. Biol.*, **86**, 40–54.

THIELE, C.J., COHEN, P.S. & ISRAEL, M.A. (1988*a*). Regulation of c-myb expression in human neuroblastoma cells during retinoic acid-induced differentiation. *Mol. Cell. Biol.*, **8**, 1677–1683.

THIELE, C.J., DEUTSCH, L.A. & ISRAEL, M.A. (1988*b*). The expression of multiple proto-oncogenes is differentially regulated during retinoic acid induced maturation of human neuroblastoma cell lines. *Oncogene*, **3**, 281–288.

TSAI-PFLUGFELDER, M., LIU, L.F., LIU, A.A., TEWEY, K.M., WHANG-PENG, J., KNUTSEN, T., HUEBNER, K., CROCE, C.M. & WANG, J.C. (1988). Cloning and sequencing of cDNA encoding human DNA topoisomerase II and localization of the gene to chromosome region 17q21-22. *Proc. Natl Acad. Sci. USA*, **85**, 7177–7181.

TSUJI, K., HAYATA, Y. & SATO, M. (1976). Neuronal differentiation of oat cell carcinoma *in vitro* by dibutyryl cyclic adenosine 3′,5′-monophosphate. *Cancer Lett.*, **1**, 311–318.

UEDA, K., CLARK, D.P., CHEN, C., RONINSON, I.B., GOTTESMAN, M.M. & PASTAN, I. (1987). The human multidrug resistance (mdr1) gene. cDNA cloning and transcription initiation. *J. Biol. Chem.*, **2**, 505–508.

WEBER, S., ZUCKERMAN, J.E., BOSTWICK, D.G., BENSCH, K.G., SIKIC, B.I. & RAFFIN, T.A. (1985). Gastrin releasing peptide is a selective mitogen for small cell lung carcinoma *in vitro*. *J.Clin. Invest.*, **75**, 306–309.

WEVER, U.M., LIOTTA, L.A., JAYE, M., RICIA, G.A., DROHAN, W.N., CLAYSMITH, A.P., RAO, C.N., WIRTH, P., COLIGAN, J.E., ALBRECHTSEN, R., MUDRYI, M. & SOBEL, M.E. (1986). Altered levels of laminin receptor mRNA in various human carcinoma cells that have different abilities to bind laminin. *Proc. Natl Acad. Sci. USA*, **83**, 7137–7141.

ZWELLING, L.A., ESTEY, E., SILBERMAN, L., DOYLE, S. & HITTELMAN, W. (1987). Effect of cell proliferation and chromatin conformation on intercalator-induced, protein-associated DNA cleavage in human brain tumor cells and human fibroblasts. *Cancer Res.*, **47**, 251–257.